# Inorganic Nanozyme with Combined Self-Oxygenation/Degradable Capabilities for Sensitized Cancer Immunochemotherapy

**DOI:** 10.1007/s40820-019-0305-x

**Published:** 2019-09-09

**Authors:** Jie Wang, Lan Fang, Ping Li, Lang Ma, Weidan Na, Chong Cheng, Yueqing Gu, Dawei Deng

**Affiliations:** 1 Department of Pharmaceutical Engineering and Department of Biomedical Engineering, School of Engineering, China Pharmaceutical University, Nanjing, 211198 People’s Republic of China; 2 Institute of Chemistry and Biochemistry, Freie Universität Berlin, Takustrasse 3, 14195 Berlin, Germany; 3 State Key Laboratory of Polymer Materials Engineering, College of Polymer Science and Engineering, Sichuan University, Chengdu, 610065 People’s Republic of China; 4 State Key Laboratory of Natural Medicines, National R&D Center for Chinese Herbal Medicine Processing, China Pharmaceutical University, Nanjing, 211198 People’s Republic of China

**Keywords:** Inorganic nanozyme, Self-oxygenation nanoreactor, Biodegradable nanomedicine, Immunochemotherapy, Cancer treatment

## Abstract

Recently emerged cancer immunochemotherapy has provided enormous new possibilities to replace traditional chemotherapy in fighting tumor. However, the treatment efficacy is hampered by tumor hypoxia-induced immunosuppression in tumor microenvironment (TME). Herein, we fabricated a self-oxygenation/degradable inorganic nanozyme with a core–shell structure to relieve tumor hypoxia in cancer immunochemotherapy. By integrating the biocompatible CaO_2_ as the oxygen-storing component, this strategy is more effective than the earlier designed nanocarriers for delivering oxygen or H_2_O_2_, and thus provides remarkable oxygenation and long-term capability in relieving hypoxia throughout the tumor tissue. Consequently, in vivo tests validate that the delivery system can successfully relieve hypoxia and reverse the immunosuppressive TME to favor antitumor immune responses, leading to enhanced chemoimmunotherapy with cytotoxic T lymphocyte-associated antigen 4 blockade. Overall, a facile, robust and effective strategy is proposed to improve tumor oxygenation by using self-decomposable and biocompatible inorganic nanozyme reactor, which will not only provide an innovative pathway to relieve intratumoral hypoxia, but also present potential applications in other oxygen-favored cancer therapies or oxygen deficiency-originated diseases.

**Electronic supplementary material:**

The online version of this article (10.1007/s40820-019-0305-x) contains supplementary material, which is available to authorized users.

## Highlights

- Self-oxygenation/degradable nanozyme reactors with core–shell structure were fabricated for relieving tumor hypoxia.
- Intratumoral hypoxia alleviation and sensitization of immunochemotherapy using as-prepared nanozyme reactors was demonstrated in B16F10 melanoma tumor.

**Electronic supplementary material:**

The online version of this article (10.1007/s40820-019-0305-x) contains supplementary material, which is available to authorized users.

## Graphical abstract

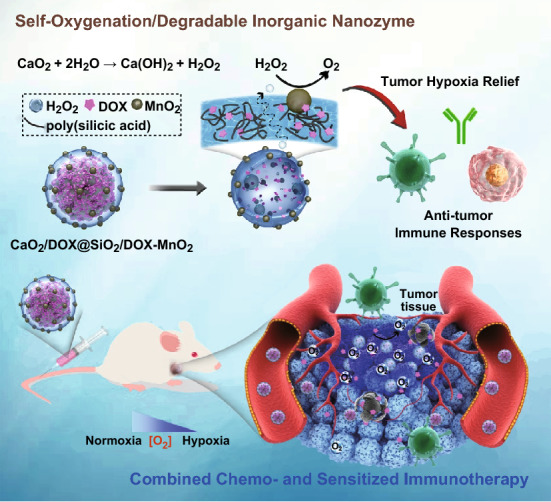

## Introduction

Cancer immunochemotherapy that integrates chemotherapeutic and immune-modulating agents has attracted increasing attentions due to the synergistically enhanced antitumor effects [1]. However, the development of immunochemotherapy is hampered by the lack of a nanoplatform that can effectively and simultaneously achieve both therapeutic goals in tumor tissues [2]. In the emerging immunochemotherapy for cancer treatment, it is often encountered that the immune system fails to respond to the tumor cells [3], even when a large number of antitumor T cells are present [4]. It has been clear that the TME, featured with low pH values, insufficient blood flow, overproduced peroxide and hypoxia, plays a vital role in such failures for tumor immunosuppression [5–7]. Among these characteristics, the hypoxia has been reported as one of the most unfavorable features that tend to suppress the immunotherapeutic efficiency [8–10]. In many in vivo situations, the activation and effector function of antitumor T cells can be diminished in the hypoxic TME, which thus protect tumor cells from being eliminated via immune system [11]. A dominant pathway in such immunosuppression is the hypoxia-adenosinergic signaling [12], because of which the immune-cell function will be negatively regulated, the production of effector cytokines such as interferon-γ (IFN-γ) will be reduced and inhibitors of this signaling such as A2AR antagonist and extracellular adenosine-degrading drugs have been used as adjuvant to compromise the hypoxia-induced tumor protection. Hence, modulating the unfavorable hypoxia characteristic of TME has been considered as an effective and clinically valuable strategy for improving tumor immunotherapy [13, 14].

To overcome the tumor hypoxia-induced immunosuppression, different strategies have been proposed recently to enhance the tumor oxygenation, for instance by increasing the intratumoral blood flow via normalization of tumor vasculatures [15], or using perfluorocarbon to deliver oxygen to hypoxic tumor tissue [16]. A direct and robust approach proposed by Sitkovsky’s group was to provide supplementary oxygen, in which tumor-bearing mice were placed in chambers containing 60% oxygen for respiratory hyperoxia [17–19]. This treatment effectively weakened hypoxia and alleviated immunosuppression, resulting in strong tumor rejection [18, 19]. Meanwhile, it has been proved that the tumor cells can produce excessive amounts of H_2_O_2_ within the TME by the overexpressed superoxide dismutase [20], which thus has been utilized for producing O_2_ to relieve the tumor hypoxia [21]. The development of nanotechnology offers new opportunities to address the shortcomings of conventional designs [22–28]; inorganic nanomaterials with catalase mimetic properties, such as the MnO_2_ and carbon nitride-based nanostructures, have been designed to generate oxygen in tumor tissue [29–35]. However, the concentration of endogenous H_2_O_2_ is extremely low, generally reported to be 50–100 × 10^−6^ M, [20, 36] which is too low to generate sufficient amount of oxygen for inducing satisfactory tumor hypoxia relief [20]. Very recently, the tumor oxygenation by delivering exogenous H_2_O_2_ and the subsequent catalase-triggered H_2_O_2_ decomposition/oxygen generation has been proposed to offer a more effective approach for tumor oxygenation [37]. However, direct delivery of H_2_O_2_ via liposomes or H_2_O_2_-related particles possess several intrinsic drawbacks, such as difficulty in preparation and storage, relatively low stability after injection, and hard to control the releasing speed of H_2_O_2_ and maintain the activity of catalase, which will undoubtedly hamper its practical application for tumor hypoxia. Therefore, fabricating more robust and facile alternative with enhanced physiological stability and lowered cost is highly desirable and necessary.

Herein, we report a self-oxygenation/degradable inorganic nanozyme reactor (NR) that can effectively relieve tumor hypoxia for sensitized cancer immunochemotherapy. The core of nanozyme reactor is composed of CaO_2_/doxorubicin (DOX), and the shell consists of degradable SiO_2_/DOX-MnO_2_; thus, this whole nanoplatform is named as CaO_2_/DOX@SiO_2_/DOX-MnO_2_ (CDSDM) NR. CaO_2_ can be gradually hydrolyzed to generate H_2_O_2_, which as the “fuel” can diffuse through the shell and decompose in the presence of MnO_2_ to produce oxygen. Meanwhile, the embedded DOX in CaO_2_ core and SiO_2_ shell allows efficient chemotherapy and also facilitates the self-decomposition of the resulting SiO_2_/DOX structure. Compared with nanoparticles without CaO_2_ core that can only decompose the tumor endogenous H_2_O_2_, the CaO_2_ core-contained CDSDM NR provides much remarkable oxygenation and long-term capability in relieving hypoxia throughout the tumor tissue.

In vivo tests also validate that treatment with these CDSDM NRs can efficiently relieve hypoxia in B16F10 melanoma tumor model, thus reversing the immunosuppressive TME to favor antitumor immunities. By combining cytotoxic T lymphocyte-associated antigen 4 (CTLA-4) blockade-mediated immunotherapy with CDSDM NR treatment, strong tumor regression was observed. Further studies reveal that CDSDM NR treatment can enhance the intratumoral infiltration of antitumor CD8^+^ T cells and decrease the population of immunosuppressive regulatory T cells (Tregs). Moreover, it has been noticed that these CDSDM NRs show no obvious toxicity. Therefore, our TME-motivated self-oxygenation/degradable inorganic nanozyme reactor not only provides an innovative and facile tumor oxygenation strategy to relieve tumor hypoxia, but also holds great potential in other oxygen-favored therapies and oxygen deficiency-originated diseases, as well as in anti-pathogen treatments [38].

## Experimental

### Synthesis of CSDM and CDSDM NRs

Preparation of CaO_2_ and CaO_2_/DOX cores: The method was derived from the gas diffusion-controlled preparation of CaCO_3_ NPs. Typically, 50 mg of CaCl_2_·2H_2_O was dissolved in 50 mL of ethanol in a beaker, into which 0.2 mL of 30% H_2_O_2_ solution was added. The beaker was then covered by parafilm with a few holes and left in a desiccator at 35 °C together with a glass bottle containing 2 mL of ammonia. After 2 h of reaction, white colloid was obtained in the beaker. The product was centrifuged and washed with ethanol for further use. For preparation of CaO_2_/DOX cores, 5 mg of doxorubicin hydrochloride (DOX·HCl, 98%) was added into the solution containing CaCl_2_ and H_2_O_2_ before incubating with ammonia.

Encapsulation of self-decomposable SiO_2_/DOX shell: 4 mL of about 2.5 mg mL^−1^ of CaO_2_ core or CaO_2_/DOX solution was dissolved into 50 mL absolute ethanol, and then 15 mg of polyvinyl pyrrolidone (PVP, MW ~ 58,000, k29–32) was added. Under stirring, 30 μL of 2 M NaOH solution and 15 mg of DOX·HCl are sequentially added. 0.1 mL of tetraethyl orthosilicate (TEOS, 99.99%) was then added. After 24 h of reaction in dark, the solution was centrifuged at 8000 rpm and washed with water three times and dispersed in water for further use.

Preparation and adsorption of CSDM and CDSDM NRs: Ultra-small MnO_2_ nanodots were first prepared according to previous reports with certain modification [30]. Briefly, 70 mg of KMnO_4_ (≥ 99.5%) and 55 mg of poly(allylamine hydrochloride) (PAH, MW ~ 15,000, 98%) were separately dissolved into 10 mL of deionized water. Under stirring, the KMnO_4_ solution was added dropwise into the PAH solution. After 15 min of reaction, 0.75 mL of pH 6.5 phosphate buffer saline (PBS) was added to 10 mL of the brown solution to disturb the stabilization by PAH. The obtained mixture was centrifuged at 10,000 rpm for 5 min and washed with water. 5 mL of about 1 mg mL^−1^ of SiO_2_/DOX shell-encapsulated NP solution was mixed with 0.2 mL of about 0.5 mg mL^−1^ MnO_2_ nanodot solution under stirring, and CDSDM or CSDM NRs were obtained via charge attraction.

### Characterizations

UV–Vis spectrophotometer (Shimadzu UV-2550) and fluorescence spectrophotometer (Edinburgh FS5) were used to obtain absorption and photoluminescence (PL) spectra, respectively. Transmission electron microscope (TEM) images were taken using a HT7700 (Hitachi) transmission electron microscope. Selected area diffraction and elemental mapping were acquired using a JEM 2100 (JEOL) transmission electron microscope. X-ray photoelectron spectrometry (XPS) assay was performed using a PHI 5000 VersaProbe (UIVAC-PHI, Japan) spectrometer.

### Oxygen Generation and DOX Release

Oxygen generation by NRs was detected based on the decrease in fluorescence intensity of tris(4,7-diphenyl-1,10-phenanthroline) ruthenium dichloride (Ru(dpp)_3_Cl_2_, 98%) in the presence of oxygen. Under N_2_ atmosphere, CDSDM NRs (~ 2 mg mL^−1^) were mixed with ethanolic solution of Ru(dpp)_3_Cl_2_ (40 μM) and incubated for different durations. At scheduled time points, the solution was centrifuged (8000 rpm, 3 min) and the supernatant was subjected to fluorescence detection with the tube sealed. CDSDM NR solution without Ru(dpp)_3_Cl_2_ was independently tested to exclude the influence of DOX fluorescence. DOX release from CDSDM NRs was measured by dialyzing with filter of 1000 Da cut-off. For in vitro hypoxia level evaluation, B16F10 cells were cultured for 4 h in 0.5 L rectangular jar sealed with a pouch, and then 0.2 mL of PBS, hDSDM (1.2 mg mL^−1^) or CDSDM (2.5 mg mL^−1^) NRs was added, followed by pimonidazole. After 4 h of incubation, cells were washed and FITC-conjugated anti-pimonidazole (Hypoxyprobe) was added and further co-incubated for 1 h. Cell imaging was performed with a laser confocal scanning microscope (LCSM, Olympus Fluoview 1000).

### Evaluation of In Vivo NR Distribution

In vivo imaging and histological section imaging were performed to study the in vivo NR distribution. For in vivo imaging, CDSDM NRs were intravenously injected into B16F10 tumor-bearing mice. IVIS In Vivo Imaging System (PerkinElmer) was used to monitor the NR distribution in a mouse based on the fluorescence of DOX (*λ*_ex_/*λ*_ex_ = 500/610 nm). After different time post-injection, mice were sacrificed and major organs and tumor were collected for imaging and then sliced into sections for histological imaging using LCSM. All in vivo experiments involving mice were approved by the Jiangsu Association for Laboratory Animals and the Department of Science and Technology of Jiangsu Province.

### Evaluation of Intratumoral Hypoxia

Balb/c mice with established B16F10 tumors were intravenously injected with saline, hDSDM NPs (0.2 mL of 1.2 mg mL^−1^) or CDSDM NRs (0.2 mL of 2.5 mg mL^−1^). Hypoxyprobe-1 Green kit was used to detect hypoxia. Eight-hour post-injection, pimonidazole (2 mg per mouse) was intravenously injected. 1 h later, tumors were dissected and cryo-sectioned, which were then co-incubated with anti-pimonidazole antibody. Cell nuclei were stained using 4′,6-diamidino-2-phenylindole (DAPI). Photoacoustic imaging and 3D cell culture model were also employed for the evaluation of hypoxia relief. Endra Nexus 128 PA imaging system was used for photoacoustic imaging, which was based on the different peak absorptions of oxyhemoglobin (~ 850 nm) and deoxyhemoglobin (~ 760 nm). Mice were kept anaesthetized so that the same section of each tumor tissue (about 3 mm from tumor surface) could be imaged for better comparison. Imaging was performed at 760 and 850 nm at each time point within 2 h. Three-dimensional tumor spheroids (400–500 μm, from KeyGen Co. Ltd., Nanjing) were co-incubated with different agents for 6 h, and pimonidazole hydrochloride was then added for another two of co-incubation. Then spheroids were cryo-sectioned and sequentially stained with anti-pimonidazole antibody and DAPI.

### Detection of Extracellular Adenosine and Cytokines

Tumor tissues excised after different treatments were firstly digested into single-cell suspensions. Erythro-9-(2-hydroxy-3-nonyl) adenine was added into the filtrates to prevent the degradation of adenosine. The resulting mixture was then centrifuged at 10,000 rpm for 10 min. The supernatants were then filtered with a 3000 Da cut-off filter, and the filtrate was freeze-dried and re-dissolved by water of 1/100 of initial volume. Adenosine measurement was carried out using high-performance liquid chromatography (Agilent 1260) (mobile phases: methanol and 10 mM KH_2_PO_4_, v/v of 1/9). For cytokine detection, serum samples were obtained from mice after above treatments, and mouse interferon γ (IFN-γ, Elabscience) and mouse tumor necrosis factor α (TNF-α, Elabscience) Elisa kits were used.

### Antitumor Treatment with NRs and CTLA-4 Blockade

Mice with established B16F10 tumors (~ 60 mm^3^) were randomly divided into five groups. The closely monitored treatment period is 20 days, during which mice were treated with saline, or saline and CTLA-4 blockade, or CSDM NRs, or CDSDM NRs, or hDSDM NPs and CTLA-4 blockade, or CDSDM NRs and CTLA-4 blockade, or CDSDM NRs + CSDM NRs (adaptive administration) and CTLA-4 blockade. In adaptive administration, CDSDM NRs were given firstly, which was replaced by CSDM NRs if tumor size reduced by 10% or more compared to the last measurement; CDSDM NRs would not be reused until tumor size increased at least 10% compared to the last measurement. Tumor size was calculated based on the formula: width^2^ × length/2. Saline, NPs (0.2 mL of 1.2 mg mL^−1^) and NRs (0.2 mL of 2.5 mg mL^−1^) were given every 2 days, while CTLA-4 blockade (20 μL) was given every 4 days. During the treatment, the tumor volume, body weight and body condition (mobility, food taking, palpable infection, etc.) were closely monitored.

### Western Blot

Mice with established B16F10 tumor were administrated with saline, hDSDM NRs (0.2 mL of 1.2 mg mL^−1^) or CDSDM NRs (0.2 mL of 2.5 mg mL^−1^). 24 h later, the tumors were excised and digested, and the single-cell suspensions were put through a 70 μm strainer. Cells were further digested in lysis buffer containing proteinase inhibitor. Lysates were centrifuged at 10,000 rpm for 5 min at 4 °C to collect the supernatant. Protein concentration was analyzed via BCA protein assay reagent. After separation with 12% SDS-PAGE, the proteins were transferred to nitrocellulose membrane. After further incubation with 5% nonfat milk powder solution, membrane was incubated with primary antibodies including anti-mouse CD39 and anti-mouse CD73 (Santa Cruz) overnight, and horseradish peroxidase-conjugated secondary antibodies were then incubated for 1 h. Enhanced chemiluminescence reagent was used to visualize the bands.

### Immunofluorescence Staining

Tumor tissues excised after 4 days of the indicated treatments in main text were embedded by OCT compound and sectioned into 10 μm slices. Shallow tissue was defined as about 0–1.5 mm from surface, while deep tissue was > 2 mm from surface. (The average diameter of tumor was about 4–6 mm.) After fixed with acetone/methanol (1/1 in v:v), FITC-conjugated anti-HIF-α and Alexa680-conjugated anti-CD8 antibodies (Santa Cruz) at a concentration of 1:100 were used for staining. Excitation of CD8 antibodies was carefully filtered to avoid the interference of DOX fluorescence. DAPI was used for staining nuclei.

### Flow Cytometry

For analysis of HIF-1α expression, tumor tissues were excised from mice after different treatments and homogenized with Collagenase D in 1640 cell culture media at 37 °C. The obtained single-cell suspensions were washed and fixed with Fix/Perm, and anti-HIF-1α-FITC (Santa Cruz) was co-incubated for 20 min in dark. For the analysis of intratumoral Tregs, the single-cell suspensions from different groups were filtered with 70 μm strainer and washed, and anti-CD25-Alexa Fluor 790 (Santa Cruz), anti-CD4-FITC (Santa Cruz) and anti-Foxp3-Alexa Fluor 680 (Santa Cruz) were used for surface staining in dark. For analysis of the maturation of DCs, two peripheral lymph nodes under the armpit at tumor side were isolated, and the obtained single-cell suspensions were stained with anti-CD80-FITC and anti-CD60-Alexa Fluor 680 (Santa Cruz) in dark. A BD FACSCalibur flow cytometer was used in flow cytometry.

### Statistics

SPSS Statistics 19 was used for data analysis including standard deviation and significance test. Student’s *t* test was used for the comparison of volume or concentration statistics between the test group and each of other groups.

## Results and Discussion

### Synthesis and Properties of Oxygen-Self-Produced CDSDM NRs

**Fig. 1:**
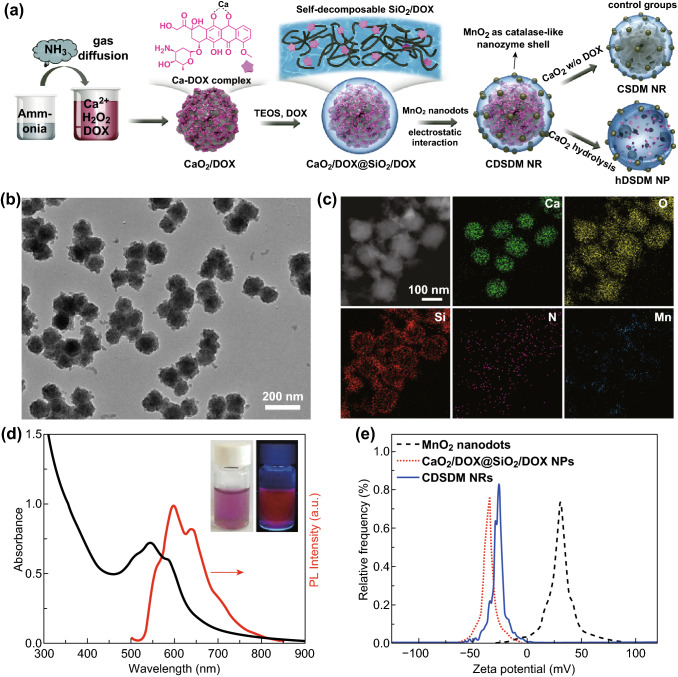
**a** Schematic illustration of the preparation of CDSDM NRs. For the control groups, the CaO_2_@SiO_2_/DOX-MnO_2_ is named as CSDM NRs; after completely hydrolyzing CaO_2_ core in the CDSDM NRs, the hollow nanoparticle is named as hDSDM NPs. **b** Representative TEM image and **c** elemental mapping of CDSDM NRs. **d** UV–Vis absorption and photoluminescence spectra of CDSDM NRs. Insets are photographs of CDSDM NRs taken under (left) room light or (right) 365 nm light irradiation. **e** Zeta Potential of MnO_2_ nanodots, CaO_2_/DOX@SiO_2_/DOX NPs and CDSDM NRs

The synthetic procedure of CDSDM NRs is illustrated in Fig. 1a; the core of NR is composed of self-decomposable CaO_2_/DOX, and the shell consists of biodegradable SiO_2_/DOX-MnO_2_. DOX was incorporated into silica shells to enable the shell self-decomposition, and was further embedded into CaO_2_ cores to improve the DOX loading capacity. Particularly, the CaO_2_ cores were prepared via a gas diffusion method (Fig. S1) and showed monodispersed spherical morphology (Fig. S2). High-resolution transmission electron microscope (HRTEM) and selected area electron diffraction (SAED) results demonstrated the CaO_2_ characteristic crystalline. DOX could be readily incorporated into CaO_2_ NPs due to the formation of Ca-DOX complex, despite the more amorphous crystalline of the resulting produces. Unlike the previously reported H_2_O_2_-filled polymersome used for reactive oxygen species-mediated therapy [39], here, the generation of H_2_O_2_ (hydrolysis of CaO_2_) was sustained and preferred in the acidic TME (pH ~ 6.5) and endosome (pH ~ 5.5) (Fig. S3), favoring the constant and specific oxygen supply to tumor tissue. CaO_2_ cores could not be dispersed in water, while the encapsulation of SiO_2_ shell allowed the CaO_2_ cores to be stably dispersed in aqueous media. Successful SiO_2_ shell encapsulation needs to control the water concentration in the ethanol solution containing CaO_2_ cores and tetraethyl orthosilicate (TEOS, SiO_2_ precursor) because high water concentration would lead to aggregation of CaO_2_ cores. Hence, concentrated NaOH solution instead of ammonia was used to catalyze the TEOS hydrolysis. Incorporation of DOX into shells was achieved by introducing DOX into the SiO_2_ shell growth media. With the constant DOX release, the shell would collapse and decompose into pieces that can be excreted via renal system [40, 41]. After the optimization of shell thickness (Fig. S4), a 15 nm shell was selected for the subsequent study.

Ultra-small (~ 1.2 nm) MnO_2_ nanodots were prepared according to the previous reports with certain modification (Fig. S5) [30], and were adsorbed onto the shells via charge attraction, after which the NR zeta potential increased from − 35 mV to about − 26 mV. Considering the high catalytic efficacy of MnO_2_ toward H_2_O_2_, the adsorbed amount of MnO_2_ nanodots was limited to this extent to ensure the resulting NRs could be readily dispersed in water without flocculation in at least 24 h (Fig. S6). The resulting CDSDM NRs were about 125 nm in diameter (Fig. 1b). Energy-dispersive X-ray spectroscopy (EDS) mapping (Fig. 1c) shows the expected elements including Ca (from CaO_2_ cores), Si (from SiO_2_ shells), O (from cores and shells), N (from DOX) and Mn (from MnO_2_ nanodots). UV–Vis absorption and photoluminescence spectra (Figs. 1d, S7) show the DOX characteristic absorption and luminescence properties of CDSDM NRs. X-ray photoelectron spectroscopy (XPS) pattern (Fig. S8) also detected these elements, in which the relative weak Ca signal was ascribed to the limited detection depth of XPS (less than 10 nm) than the typical shell thickness (15 nm), and further demonstrated the core/shell structure of the CDSDM NRs. The Ca/Si/Mn ratio in NRs measured using inductively coupled plasma optical emission spectrometry (ICP-OES) was about 1:0.91:0.1. Zeta potential of NRs in water is shown in Fig. 1e.

**Fig. 2:**
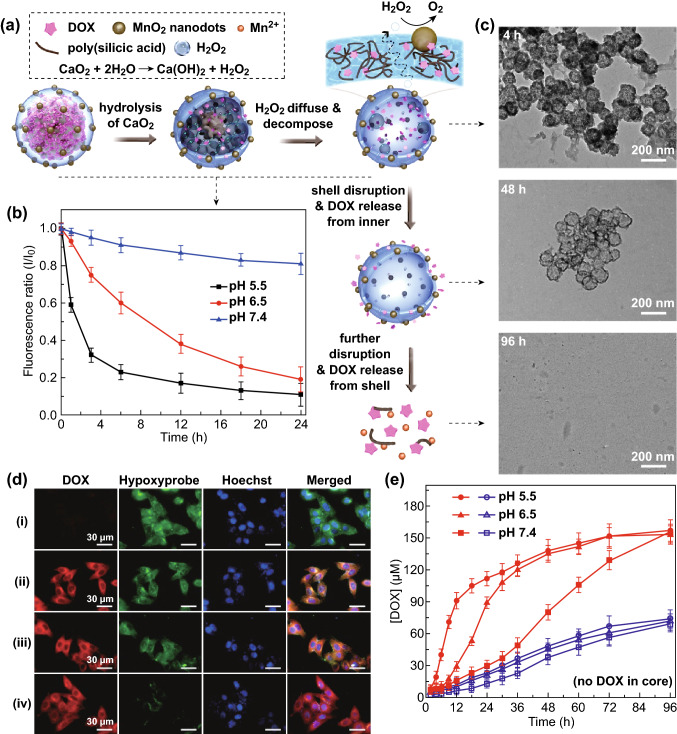
**a** Schematic illustration of the processes of oxygen generation, DOX release and decomposition of NRs. **b** The measurement of oxygen release process of CDSDM NRs, shown as the ratio of fluorescence intensity of Ru(dpp)_3_Cl_2_ (an oxygen indicator) after incubated with NRs for different durations (*I*) to that before incubation (*I*_0_). **c** TEM images of the decomposition process of CDSDM NRs after incubated in pH 5.5 PBS for different durations. **d** Representative CLSM images of B16F10 cells from hypoxia cultivation after co-incubated with (1) PBS, (2) hDSDM NPs, (3) CDSD (without MnO_2_ nanodots) NPs and (4) CDSDM NRs for 4 h. Hypoxia level was detected using Hypoxyprobe. **e** DOX release from CDSDM NRs and CSDM NRs in PBS of different pH values

Incubating CDSDM NRs in phosphate buffer saline (PBS) led to the hydrolysis of CaO_2_ into H_2_O_2_, which diffused through the permeable SiO_2_ shell and decomposed in the presence of MnO_2_ nanodots to provide oxygen (Fig. 2a). The hydrolysis of CaO_2_ cores was faster at lower pH (Fig. S3), thus facilitated the controlled oxygen supply in acidic TME and endosome, as shown in Fig. 2b. Incubating NRs in pH 5.5 PBS for about 4 h led to complete decomposition of cores, while the remained products were hollow nanoparticles (Fig. 2c, denoted as hDSDM NPs) with about 48% of the weight of whole NRs. The similar zeta potential suggested that MnO_2_ nanodots were basically retained in these hDSDM NPs. Due to the incorporation of DOX molecules, the cross-linking degree of silica matrix (products of TEOS hydrolysis) would be lowered due to the steric effect of DOX molecules. In aqueous media where DOX is soluble, the constant release of DOX from the silica will trigger the silica shell disruption and finally lead to the complete fragmentation into poly(silicic acid) that can be excreted via renal system (Fig. 2c) [41]. MnO_2_ nanodots were also decomposed after about 24 h of incubation, in accordance with previous report [29]. The self-decomposition capability of NRs was believed to be important for reducing systemic toxicity when used in vivo.

Incubating hypoxic B16F10 cells with CDSDM NRs for 4 h, the cellular hypoxia was effectively reconditioned (Fig. 2d) due to the constant oxygen supply by CDSDM NRs. In obvious comparison, the hDSDM NPs exhibited no such effect, suggesting the importance of CaO_2_ cores in providing sufficient oxygen; CDSD (CaO_2_/DOX@SiO_2_/DOX) NP treatment also showed no obvious hypoxia relief, which should be due to the limited H_2_O_2_ decomposition for oxygenation without MnO_2_ nanodots. The cytoplasmic distribution of the main DOX fluorescence suggested that DOX might be trapped inside shells at the beginning despite the decomposition of CaO_2_, because otherwise substantial DOX would be accumulated in nuclei. This was confirmed by analyzing the release profile via UV–Vis absorption measurement that the DOX release was lagged much behind the oxygen generation (Fig. 2b, e). Meanwhile, lower pH allowed faster DOX release. The DOX release from SiO_2_ shells was found to be more sustained by analyzing CSDM NRs (absorption spectra shown in Fig. S9) that contained no DOX in CaO_2_ cores, in line with the lower decomposition rate of SiO_2_ shells than CaO_2_ cores. In vitro antitumor capabilities of CDSDM NRs, CSDM NRs and hDSDM NPs were evaluated via MTT assay (Fig. S10). The results showed that CDSDM NRs exhibited significantly higher cytotoxicity than hDSDM NPs under hypoxic condition. This was ascribed to the sensitization of chemotherapy by oxygenation, which has become a widely explored strategy for improving therapeutic efficacy of chemo drugs.

### Relief of Tumor Hypoxia and Suppression of Hypoxia-Adenosinergic Signaling

**Fig. 3:**
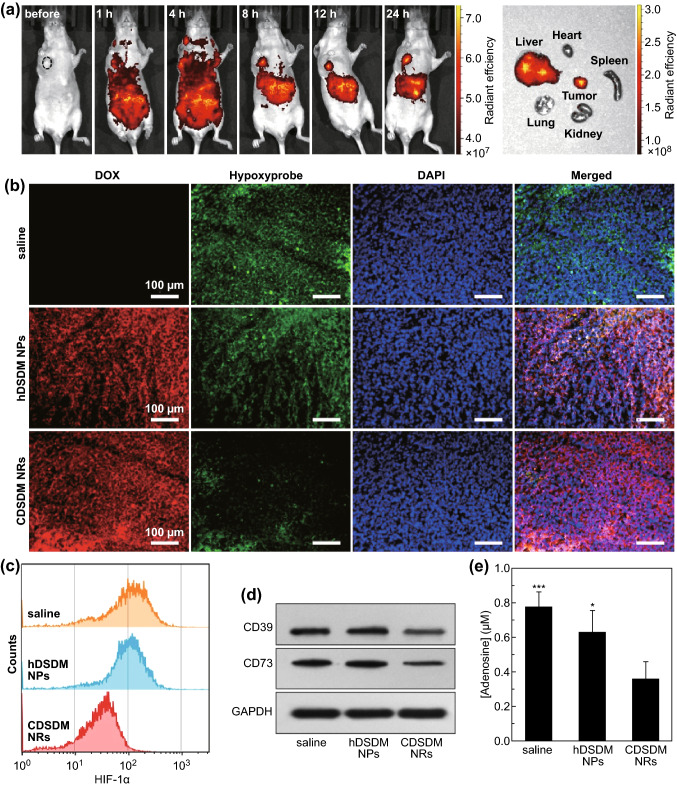
**a** In vivo fluorescence monitoring of the distribution of CDSDM NRs in B16F10 tumor-bearing mice at scheduled time points post-intravenous injection, and ex vivo fluorescence of major organs and tumor 8-h post-injection. **b** Fluorescence imaging of tumor tissue sections after receiving different treatments, in which signals include DOX fluorescence, Hypoxyprobe (hypoxia indicator) and DAPI. **c** Flow cytometric analysis of the intratumoral HIF-1α level after different treatments. **d** Western blot results of the expressions of CD39 and CD73 on intratumoral T lymphocytes after 2 days of the indicated treatments. **e** Concentrations of extracellular adenosine in tumor tissue after 2 days of the indicated treatments. *P < 0.05; ***P < 0.001 versus the last group

Lower oxygen tension in solid tumor tissue can weaken the efficacy of spontaneous or adoptive immunotherapy, in which the hypoxia-adenosinergic signaling has been known as a master pathway [12]. In this work, the capability of CDSDM NRs in reversing such immunosuppression was tested. In vivo distribution of CDSDM NRs when intravenously injected into mice-bearing B16F10 melanoma tumor under armpit was first studied. By monitoring the fluorescence signal of DOX, substantial intratumoral accumulation was observed 8-h post-injection (Fig. 3a) owing to the enhanced permeability and retention effect of tumor vasculature [42]. Similar results were observed from ex vivo imaging of excised organs and microscopic imaging of histological sections (Figs. 3a, S11). Quantitative analysis using inductively coupled plasma mass spectrometry (ICP-MS) showed up to 23% of Si accumulation in tumor 8-h post-injection (Fig. S12). Next, intratumoral hypoxia level was evaluated using hypoxia marker Hypoxyprobe. As shown in Fig. 3b, the intratumoral hypoxia was clearly alleviated even after one dose of CDSDM NR administration, in contrast to hDSDM NP treatment. Three-dimensional cell culture model (Fig. S13) and photoacoustic imaging (Fig. S14) were also employed to validate the hypoxia alleviation, and the results provided further demonstration of the superior hypoxia-relieving capability of CDSDM NRs than hDSDM NPs. Although the MnO_2_ nanodots on hDSDM NPs can catalyze the decomposition of H_2_O_2_, which is relatively abundant in tumor tissue [29, 43], they seem to be much less efficient in alleviating tumor hypoxia, further underscoring the necessity of CaO_2_ cores in this work.

Next, the effect of CDSDM NR treatment on hypoxia-adenosinergic signaling was studied. Expressions of several key molecular events of the signaling including hypoxia-inducible factor-1α (HIF-1α), CD39/CD73 and adenosine were tested. Flow cytometry and western blot results showed that treatment with CDSDM NRs resulted in significantly reduced expression of HIF-1α in cells from tumor tissue (Fig. 3c) and CD39/CD73 in T cells (Fig. 3d), respectively, compared with the hDSDM group. HIF-1α is the master regulator of hypoxic response in T cells, while the CD39/CD73 expression can be lowered by HIF-1α reduction [44, 45]; these should be ascribable to the reduced CD39/CD73 expression in T cells by hypoxia relief. HIF-1α can be rapidly degraded under normal conditions via being poly-ubiquitinated, while hypoxia (oxygen tension lower than about 3%) can drive the enzyme-mediating HIF-1α degradation nonfunctional [46]. Therefore, the reduced HIF-1α was also a further demonstration of the relief of hypoxia. The extracellular adenosine in TME also significantly decreased largely as a consequence of the reduced expression of adenosine generating CD39/CD73 (Fig. 3e). Taken together, it can be concluded that the hypoxia-adenosinergic signaling has been inhibited efficiently, through which a positive immune modulation can be expected via its effects on Tregs and cytotoxic T cells (CTL) [12].

### Strong Tumor Regression with Oxygen-Self-Generated CDSDM NRs

**Fig. 4:**
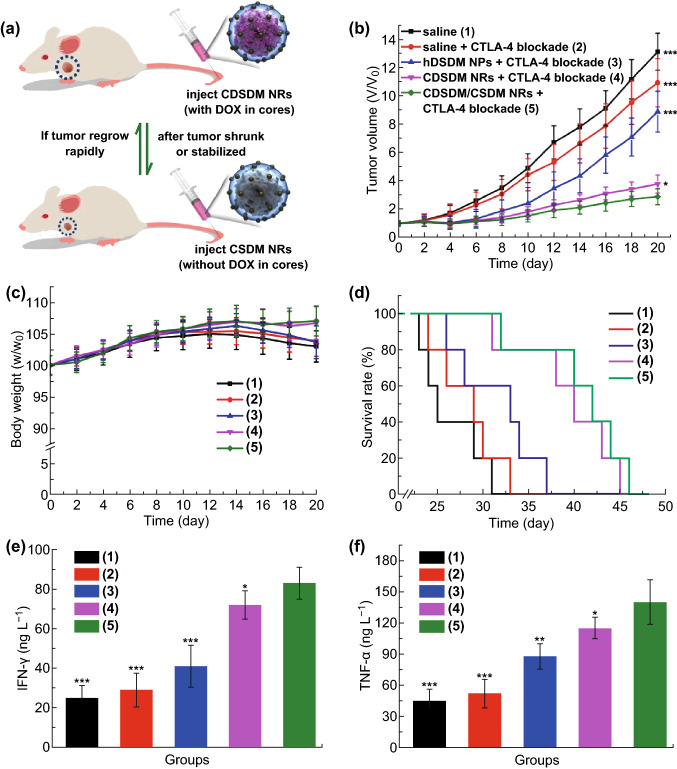
**a** Illustration of adaptive administration of CDSDM NRs and CSDM NRs. **b** Relative tumor volumes and **c** body weights of B16F10 tumor-bearing mice received with different treatments during the treatment period. **d** Survival rate of mice in each group during and after treatment. Serum levels of **e** IFN-γ and **f** TNF-α detected after 8 days of different treatments. *P < 0.05; **P < 0.01; ***P < 0.001 versus Group 5

Given the effective inhibition of hypoxia-adenosinergic signaling, one can expect a synergistic effect between NR treatment and immunotherapy. CTLA-4 blockade, a FDA approved immune checkpoint-blocking antibody for the treatment of advanced melanoma [47], was used for the eradication of B16F10 melanoma tumor in this work. To reduce the damage of DOX-containing NRs to the immune system, CSDM NRs was also used for adaptive administration with CDSDM NRs (Fig. 4a). The DOX amount in CSDM NRs was about half of that in CDSDM NRs (Table S1). Adaptive administration here means adjusting the dosage of chemotherapeutic drug according to the variation of tumor size rather than giving an invariable high dose in order to protect the immune system by chemo agent (DOX) [48, 49].

In this work, CDSDM NRs were given in initial treatment, while CSDM NRs would be used as alternatives if tumor volume decreased or increased less than 10% from the previous measurement; otherwise CDSDM NRs would be reused. In a 20-day treatment period, different agents were intravenously injected every 2 days, while CTLA-4 blocking antibody was injected intraperitoneally every 4 days. Tumor volume, body weight and survival rate in each group were monitored during and after treatment (Fig. 4b–d). Compared with the oxygen-self-produced CDSDM NRs + CTLA-4 blockade, the results showed that hDSDM NPs + CTLA-4 blockade showed much weaker antitumor efficacy (evaluated according to tumor volume, survival rate and histopathology staining of tumor tissue, Figs. 4b, d and S15, S16), although stronger than CTLA-4 blockade alone. Therefore, relief of intratumoral hypoxia should be a prerequisite for CTLA-4 blockade to exert due antitumor efficacy, considering the same DOX concentration in hDSDM NPs and CDSDM NRs. Meanwhile, adaptive administration of CDSDM/CSDM NRs showed stronger efficacy than the CDSDM NRs albeit the lower total DOX dose (Fig. S17), suggesting an immune benefit of this chemotherapy strategy [49]. In a control study using immune-deficient nude mice, the synergistic effects between CDSDM NR and CTLA-4 blockade treatment were much limited due to the attenuated cellular immunity (Fig. S19). Instead, the DOX in CDSDM NRs accounted for the observed antitumor efficacy. Therefore, it is believed that a complete immune system is a prerequisite for the sensitization of immunotherapy by CDSDM NR-induced hypoxia relief.

We also tested the immune effect in molecular level by measuring the production of typical cytokines. Measurement of the serum levels of IFN-γ and TNF-α (typical markers of cellular immune response) showed increased production in mice treated with CDSDM NRs than that with hDSDM NPs (Fig. 4e, f), which should be one of the results of the alleviation of hypoxia-adenosinergic signaling and reflected a stronger antitumor immune response [50, 51]. Adaptive administration of CDSDM and CSDM NRs further induced slight increase in cytokine production due to the more potent immune system with reduced DOX dose.

Meanwhile, no obvious reduction in body weight was observed in mice treated with CDSDM/CSDM NRs (Fig. 4c), suggesting the indistinctive systemic toxicity. The body weight loss in mice in other three groups could be ascribed to reduced food intake and fat loss due to the increased tumor burden. Hematoxylin–eosin histopathology staining results also indicated the rather limited damage of major organs caused by CDSDM/CSDM NRs (Fig. S20). Hemolysis test was also performed by incubating CDSDM NRs with red blood cells, and the hemolysis induced by CDSDM NRs at the concentration for treatment was believed to be acceptable (Fig. S21). [52] Therefore, the as-prepared oxygen-self-produced CDSDM (and CSDM) NRs were considered to be a considerably safe hypoxia-relieving nanoformulation.

### Enhanced CD8^+^ T Cell Infiltration and Suppressed Treg Development

**Fig. 5:**
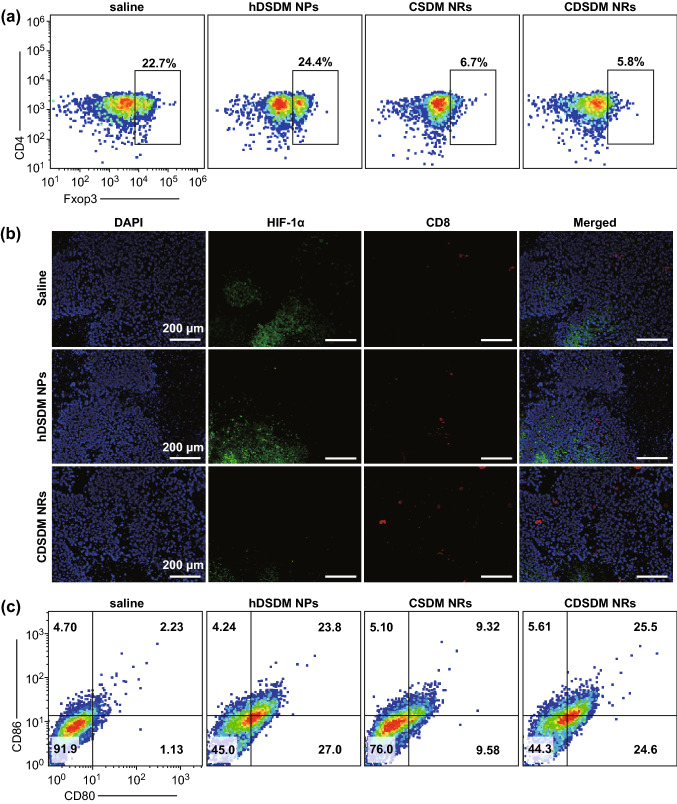
**a** Flow cytometry plots showing the percentages of intratumoral Treg (gated on CD25^+^ T cells) populations from mice after 4 days of the indicated treatments. **b** Immunofluorescence results of the co-staining of HIF-1α and CD8 to deep (> 2 mm from the surface) tumor tissues from mice received 4 days of the indicated treatments. **c** Flow cytometry plots showing the percentages of CD80^+^ and CD86^+^ matured DCs (gated on CD11c+ DCs) from the axillary lymph nodes of mice after 4 days of the indicated treatments

Tregs can diminish spontaneous or blockade-mediated antitumor immune responses via the expressions of CD39/CD73 and constitutive CTLA-4 [53]. The former could deliver negative immunomodulation via hypoxia-adenosinergic signaling, while the latter has made Treg a master target of the currently widely studied CTLA-4 blockade [47, 53]. It has been proposed that hypoxia alleviation by hyperoxia breathing could reduce the Treg population [19], and in this work, the effect of oxygen-self-produced NRs on Treg population was studied. Flow cytometric results showed that in comparison with hDSDM NPs, treatment with CDSDM or CSDM NRs resulted in a significant decrease (by a factor of ~ 4) in intratumoral Treg (CD4^+^ CD25^+^ Foxp3^+^) population (Fig. 5a). In line with the superior tumor regression using CDSDM NRs than hDSDM NPs, this suggested that Treg-related immunosuppression could be greatly alleviated in complementary manners with the inhibition of hypoxia-adenosinergic signaling and the use of CTLA-4 blockade (CTLA-4 contributes to the immunosuppressive function of Tregs). Also, it can be expected to achieve synergistic effect when combining NRs with other blocking antibodies of immune checkpoints, for instance the PD-1/PD-L1 [54].

Antitumor T cells can be repelled in hypoxic areas, and this is why endogenous developed or adoptively transferred tumor-responsive CD8^+^ T cells showed limited antitumor efficacy and were often combined with other agents such as antagonist of adenosine receptor [55]. Inspired by the observation that the recruitment of CD8^+^ T cells could be enhanced by respiratory hyperoxia [19], we tested whether the hypoxia relief with CDSDM NRs facilitated the intratumoral infiltration of antitumor CD8^+^ T cells. Tumor tissues from different groups were analyzed by the co-staining of HIF-1α and CD8, and the results suggested that in both shallow (Fig. S22) and deep (Fig. 5b) tumor tissues, treatment with CDSDM NRs resulted in reduced HIF-1α expression and enhanced intratumoral accumulation and infiltration of CD8^+^ T cells. Therefore, hypoxia alleviation with our NRs could significantly reduce the repelling of CTLs (i.e., activated CD8^+^ T cells) by tumor tissue, and as a desirable extension of the functions of CDSDM NR treatment, this was ascribed to another contribution to the enhanced tumor regression as shown in Fig. 4b.

The released DOX from CDSDM NRs, while serving as the typical chemo agent to induce apoptosis of tumor cells, was also found to be immunogenic. Incorporation of DOX into SiO_2_ shells were designed to achieve immuno-chemo-combined therapy and to enable the self-decomposition of the SiO_2_ shells; meanwhile, it has been reported that anthracyclines, in particular, DOX-treated dying or apoptotic tumor cells can serve as in situ vaccine to induce immunogenic tumor cell death in the absence of any adjuvant or co-stimulus [56, 57]. Therefore, we have explored the effect of DOX on immunogenicity by analyzing maturation of dendritic cells (DCs), which can phagocytose the DOX-treated apoptotic cells, present the neoantigens to T cells and mature during this process. DCs were isolated from axillary lymph nodes at tumor side after 4 days of different treatments, and flow cytometric results of the staining of CD80 and CD86 (typical markers of DC maturation) showed that the percentage of matured DCs was much higher in mice treated with hDSDM NPs or CDSDM NRs (Fig. 5c). The increase in median percentage in CSDM NR group was ascribed to the lower DOX concentration. Therefore, in current experimental setting, the DOX embedded in SiO_2_ shells served not only as the chemotherapeutic drug but also a vaccine primer that led to a productive immune activation, together with CTLA-4 blockade in a synergistic manner (CTLA-4 blockade can enhance the immunopermissive recognition between naive T cells and antigen-presenting mature DCs) [58].

**Scheme 1:**
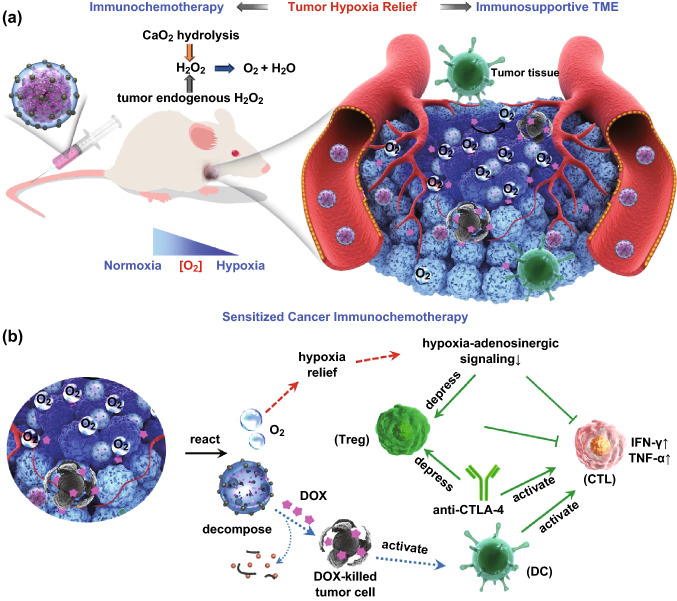
**a** Schematic illustration of the proposed roles of CDSDM NRs in tumor hypoxia relief. **b** The proposed mechanism and reaction pathways include the relief of hypoxia-induced immunosuppression (red dashed arrows), the DOX-mediated chemotherapy and immune activation (blue dotted arrows), and the synergistic actions between them and the CTLA-4 blockade (green arrows). (Color figure online)

Based on the above observations, the schematic illustration of the proposed roles of CDSDM NRs in tumor hypoxia relief is shown in Scheme 1a. Both the hydrolysis of CaO_2_ and the endogenous H_2_O_2_ within tumor contributed the abundant oxygenation via the catalase-like activity of MnO_2_ nanodots. The multiple roles of CDSDM NRs in tumor rejection are shown in Scheme 1b. Basically, the immuno-priming effects of CDSDM NRs were derived from their two capabilities, i.e., the TME-motivated oxygen-self-production, and the DOX releasing during oxygen production and NR decomposition. The former effectively relieved intratumoral hypoxia, and the resulting immune benefits included: (1) the immunosuppressive hypoxia-adenosinergic signaling was inhibited (Fig. 3b–e), and as a direct result, the production of antitumor cytokines (e.g., IFN-γ and TNF-α) by T lymphocytes increased (Fig. 4e, f). (2) The negative immunomodulation by Tregs was inhibited due to the decreased expression of CD39/CD73 (Fig. 3d) and the reduced population (Fig. 5a). These effects could promote the activation of antitumor immune responses in a complementary manner with the CTLA-4 blockade. (Treg is a master expression host for CTLA-4). (3) Due to the decreased hypoxic repelling effect, the intratumoral infiltration and accumulation of CD8^+^ T cells were improved (Fig. 5b). The released DOX from CDSDM NRs, while serving as the typical chemo agent to induce apoptosis of tumor cells, was also found to be immunogenic by inducing DC maturation (Fig. 5c) and thereby promoting T cell activation synergistically with CTLA-4 blockade.

## Conclusion

In summary, a self-oxygenation/degradable inorganic nanozyme system has been fabricated to relieve tumor hypoxia for cancer immunochemotherapy. By integrating CaO_2_ core as the oxygen-storing component, our system provides improved oxygenation and capability of relieving hypoxia compared with the oxygenation methods that only decompose the endogenous H_2_O_2_ in TME. Compared to the reported design that directly delivers H_2_O_2_, our strategy shows acid TME-preferred oxygenation and enhanced physiological stability. In vivo tests validated that the CDSDM NRs could successfully relieve hypoxia in mice-bearing B16F10 tumor, thus reversing the immunosuppressive TME to favor antitumor immune response. Consequently, the CTLA-4-mediated immunotherapy was greatly sensitized. Further studies revealed that CDSDM NR treatment also enhanced the intratumoral infiltration of CD8^+^ T cells and decreased the population of immunosuppressive Tregs. Meanwhile, the DOX in CDSDM NRs also elicited desirable maturation of DCs, further favored a productive antitumor immune response. Overall, a novel self-oxygenation/degradable nanozyme system has been proposed to improve the cancer immunochemotherapy via relieving tumor hypoxia without causing notable toxic side effects. This strategy not only shows promise in relieving tumor hypoxia and sensitizing cancer immunochemotherapy, but also holds the potential for applications in oxygen-favored cancer therapy (i.e., photodynamic and radiation therapy) and oxygen deficiency-originated diseases.

## Electronic supplementary material

Below is the link to the electronic supplementary material.

Supplementary material 1 (PDF 1418 kb)
