# Supplementary material for: Inorganic Nanozyme with Combined Self-Oxygenation/Degradable Capabilities for Sensitized Cancer Immunochemotherapy
Source: Nanomicro Lett. 2019 Sep 9;11:74. doi: 10.1007/s40820-019-0305-x (PMC7770972; doi:10.1007/s40820-019-0305-x)
Supplement: Supplementary file 1 — Supplementary material 1 (PDF 1418 kb) [file 40820_2019_305_MOESM1_ESM.pdf]

Supporting Information for

Inorganic Nanozyme with Combined Self-Oxygenation/Degradable Capabilities for Sensitized Cancer Immunochemotherapy

Jie Wang[1], Lan Fang[1], Ping Li[1], Lang Ma[2, 3], Weidan Na[1], Chong Cheng[2, 3, *], Yueqing Gu[1], Dawei Deng[1, 4, *]

[1]Department of Pharmaceutical Engineering and Department of Biomedical Engineering, School of Engineering, China Pharmaceutical University, Nanjing 211198, People's Republic of China

[2]Institute of Chemistry and Biochemistry, Freie Universität Berlin, Takustrasse 3, 14195 Berlin, Germany

[3]College of Polymer Science and Engineering, State Key Laboratory of Polymer Materials Engineering, Sichuan University, Chengdu, 610065, People's Republic of China

[4]National R&D Center for Chinese Herbal Medicine Processing, and State Key Laboratory of Natural Medicines, China Pharmaceutical University, Nanjing 211198, People's Republic of China

*Corresponding authors. E-mail: dengdawei@cpu.edu.cn (D. Deng); chong.cheng@fu-berlin.de (C. Cheng)

Supplementary Figures

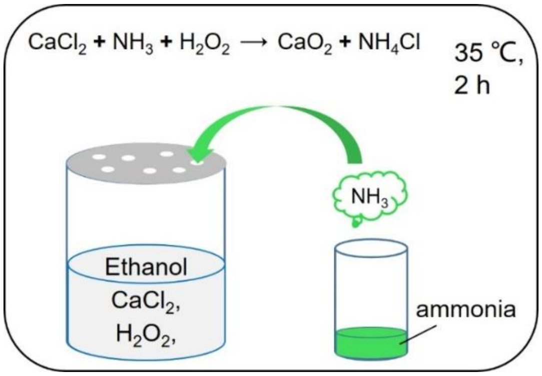

Fig. S1 Schematic illustration of the preparation of CaO_2 NPs

A beaker containing ethanol solution of $CaCl_2$, and H_2O_2 (50 mL in total) was covered by parafilm with a few pores, and were placed in a desiccator with a smaller beaker containing

1 mL of ammonia (25–28%). After 2 h of gas diffusion reaction at 35 ºC, CaO_2 cores were fabricated. During the reaction, NH_3 vapor diffused into the reaction beaker to drive the indicated reaction forward. CaO_2/DOX cores could be prepared when DOX were introduced into the reaction beaker.

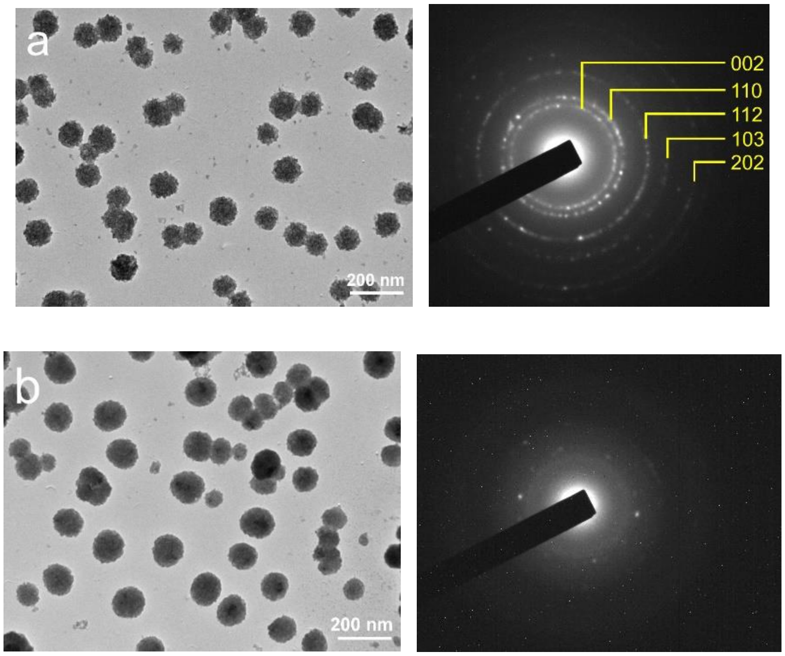

Fig. S2 TEM images and the corresponding selected area electron diffraction patterns of **a** CaO_2 cores and **b** CaO_2/DOX cores. The mean diameters were about 90 and 95 nm for CaO_2 and CaO_2/DOX cores, respectively.

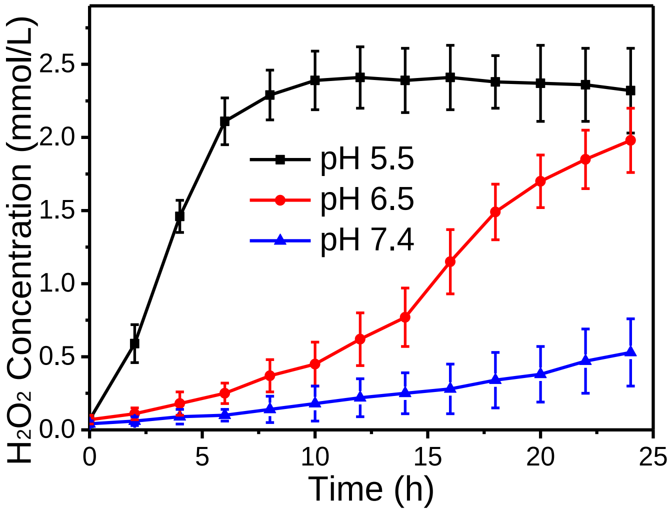

Fig. S3 H_2O_2 concentration when incubating CDSD NPs (1 mg mL^{-1}, 10 mL) in 10 mL of PBS with different pH values

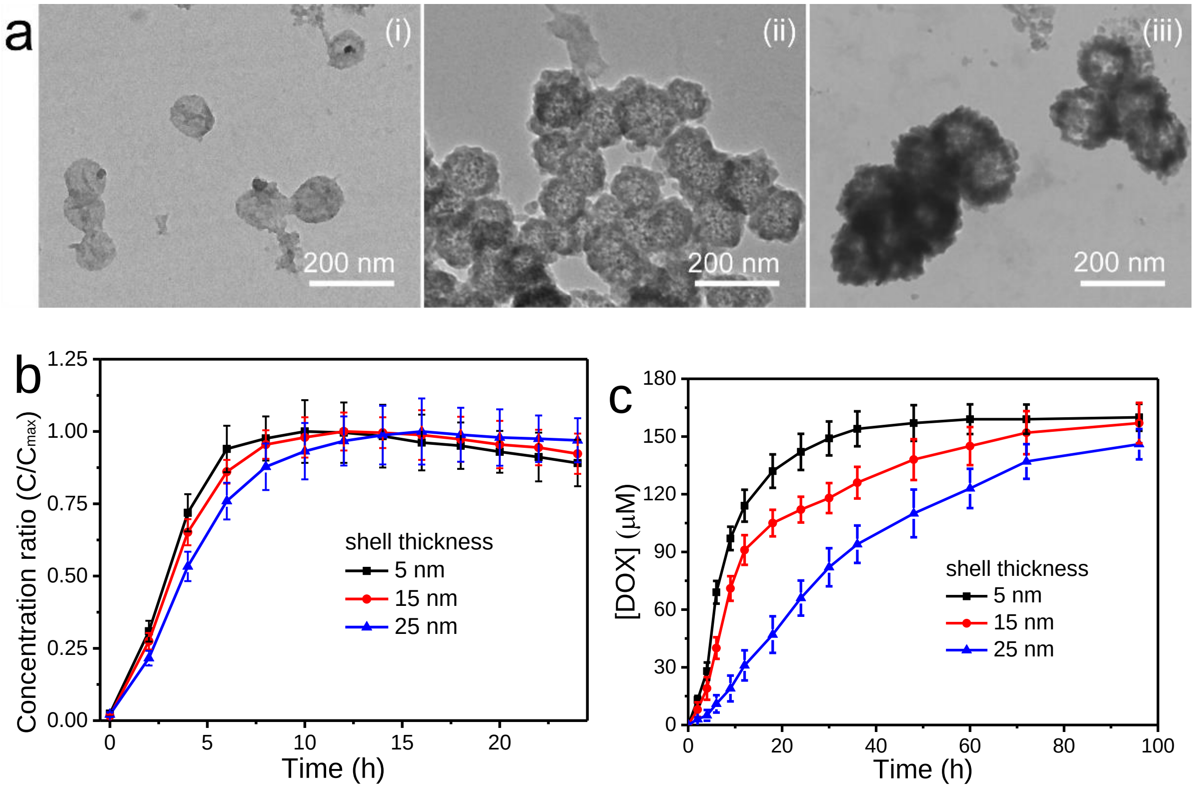

Fig. S4 a TEM images of CDSDM NRs with different shell thicknesses (i, ~5 nm; ii, 15 nm; iii, ~25 nm) after incubated in pH 5.5 PBS for 6 h. **b** H_2O_2 generation from CDSD NPs of different shell thicknesses in pH 5.5 PBS. MnO_2 nanodots were not adsorbed to allow H_2O_2 detection. Each line was normalized by the maximum concentration for better comparison of the CaO_2 hydrolysis rates. **c** DOX release from CDSDM NRs of different shell thicknesses

The results showed that all these shell thicknesses enabled the resulted NRs to be water-soluble. Meanwhile, a thicker silica shell led to slightly slower CaO_2 hydrolysis and a significantly slower DOX release from CaO_2/DOX cores. The reason should be that the limited permeability of silica shell selectively impeded the diffusion of DOX molecules. Slower CaO_2 hydrolysis could allow constant oxygenation, while slow DOX release rate is not favorable for realizing therapeutic efficacy at effective concentration. Furthermore, thicker shell led to larger particles, which would be more prone to be cleared by the reticuloendothelial system during circulation. Therefore, the shell thickness of 15 nm should be in the appropriate range for this study.

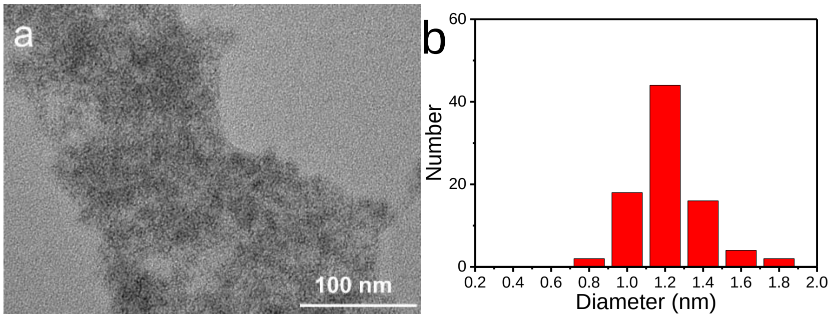

Fig. S5 a TEM image and **b** size distribution (measured using Nano Measure 1.2 software) of ultra-small MnO_2 nanodots. The mean diameter is about 1.2 nm.

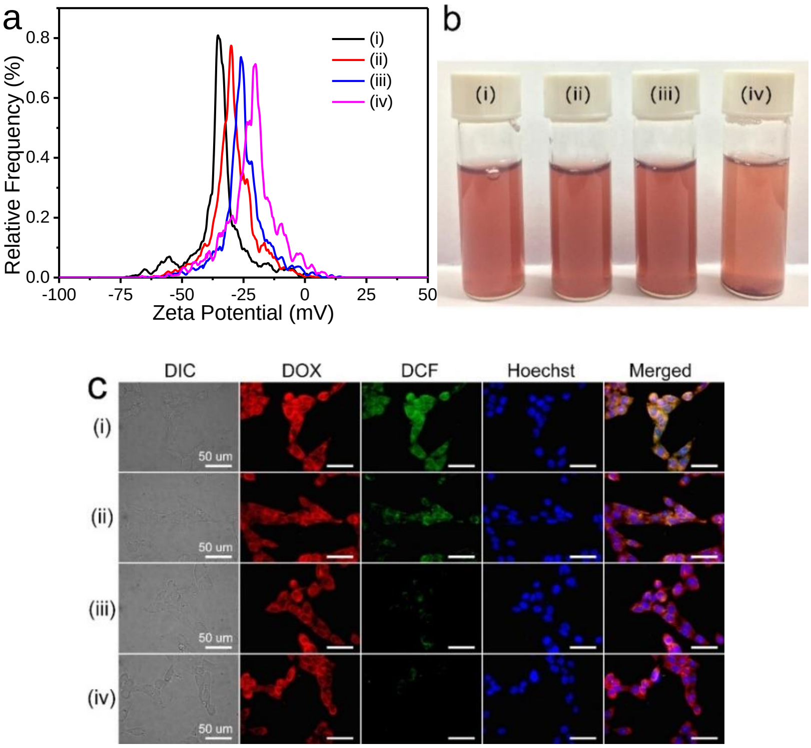

Fig. S6 a Zeta Potential of CDSDM NRs with different adsorbed amount of MnO_2 nanodots. Increasing the MnO_2 adsorption would increase the zeta potential of NRs: i, -35 mV (no adsorption); ii, -30 mV; iii, -26 mV; iv, -20 mV. **b** Photograph of the corresponding CDSDM NRs after dissolved in fetal bovine serum for 24 h. Increasing MnO_2 adsorption would disturb the stability of NR solution due to the decreased absolute value of zeta potential, as shown in the fourth vial by the flocculation of NRs with -20 mV zeta potential after 24 of standing. **c** Representative CLSM images of B16F10 cells incubated with the corresponding NRs. Green fluorescence represents the presence of reactive oxygen species (H_2O_2) detected using dichlorodihydrofluorescein (DCF)-based reactive oxygen species assay kit. CDSDM NRs with zeta potential of -26 mV were sufficient for H_2O_2 decomposition. Therefore, the absorbed MnO_2 nanodots in the NRs used in the present study (zeta potential of -26 mV) should be in the appropriate range.

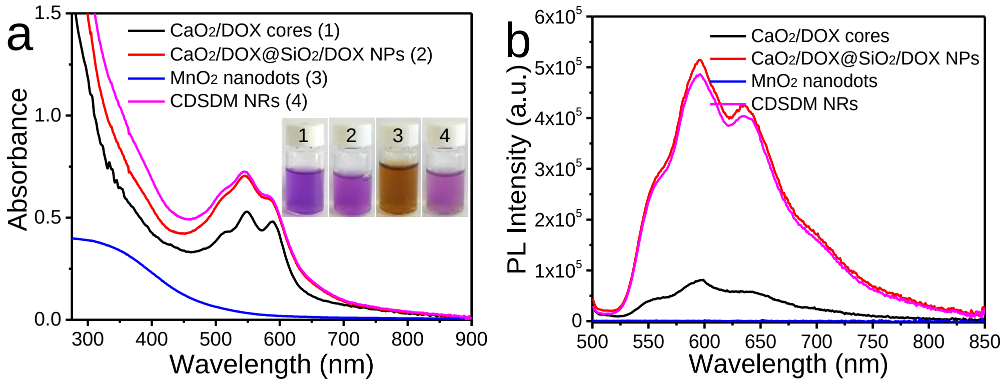

Fig. S7 a UV-Vis absorption and **b** photoluminescence spectra measurement of the key intermediates (CaO_2/DOX cores in ethanol, CaO_2/DOX@SiO_2/DOX NPs in water and MnO_2 nanodots in water) and CDSDM NRs in water

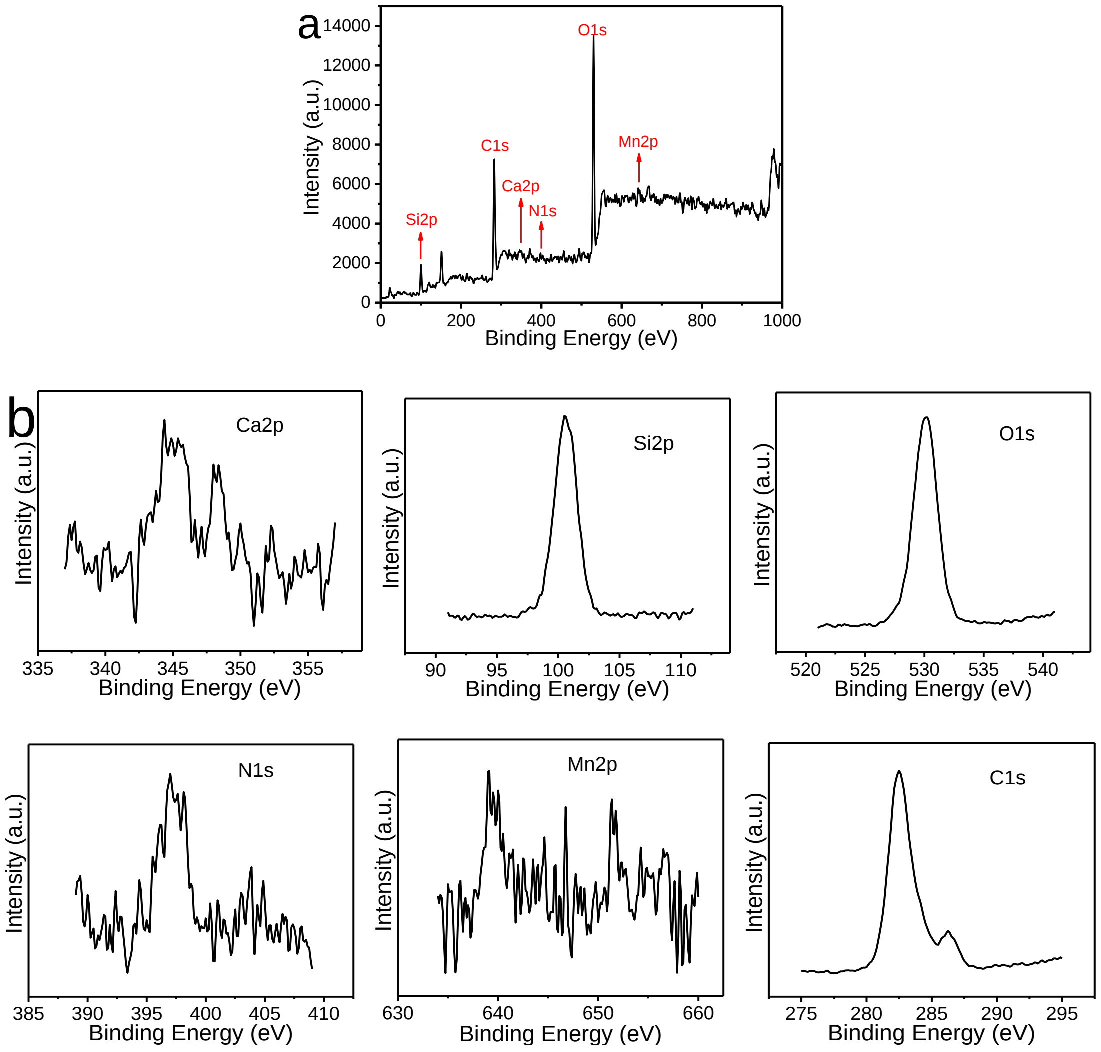

Fig. S8 XPS spectra of CaO_2/DOX@SiO_2/DOX-MnO_2 NRs: **a** full spectrum and **b** spectra of the indicated elements. All expected elements including Ca (from CaO_2/DOX cores), Si

(from SiO_2/DOX shells), O (from cores and shells), N (from DOX), Mn (from MnO_2 nanodots) and C (from DOX and capping ligands on MnO_2 nanodots) were detected with an atomic concentration ratio of 0.47/10.40/35.47/1.14/0.66/51.86. The relative weak Ca signal was ascribed to the limited detection depth of XPS (less than 10 nm) that was smaller than the typical shell thickness (15 nm) measured from TEM images.

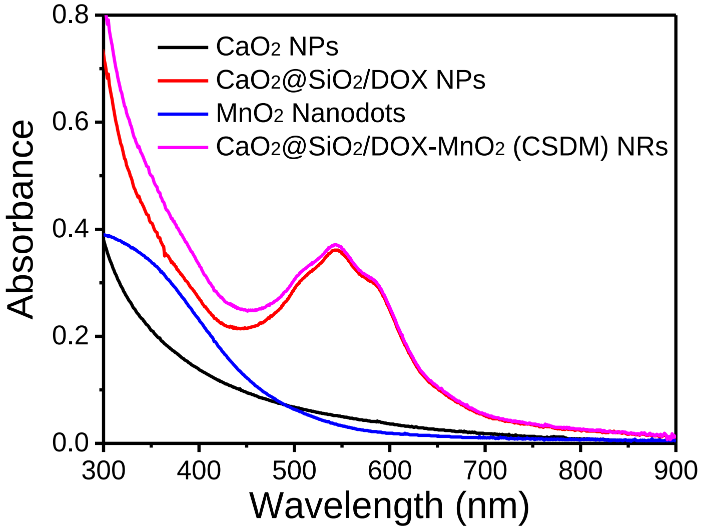

Fig. S9 Absorption spectra of CaO_2 cores (without DOX), CaO_2@SiO_2/DOX NPs, MnO_2 nanodots and CSDM NRs

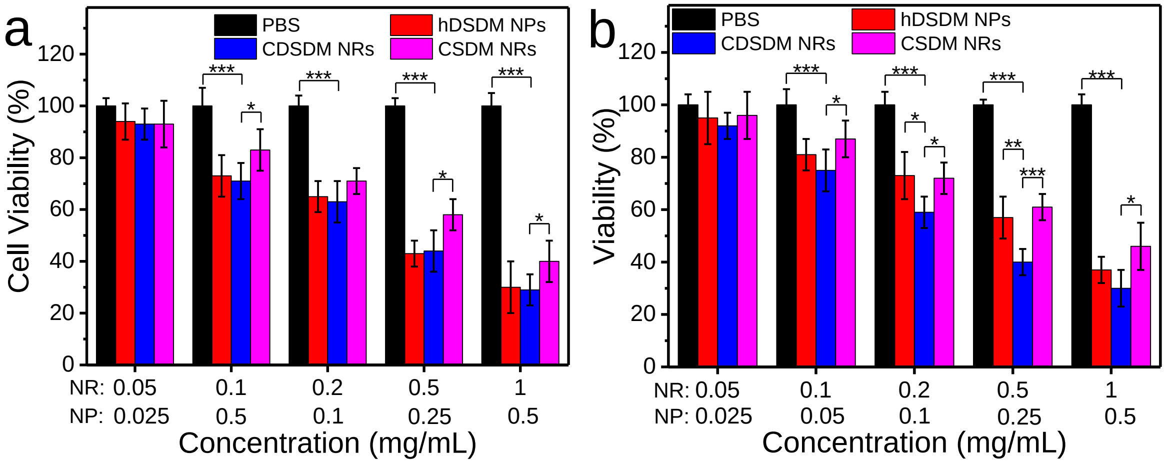

Fig. S10 B16F10 cell viabilities after treatment with different therapeutic agents at different concentrations under **a** normoxia or **b** hypoxia condition. The first line of concentration accounts for CDSDM NRs and CSDM NRs, while the second line corresponds to hDSDM NPs. This was *P<0.05, **P<0.01, ***P<0.001 versus Group 3 (CDSDM NRs)

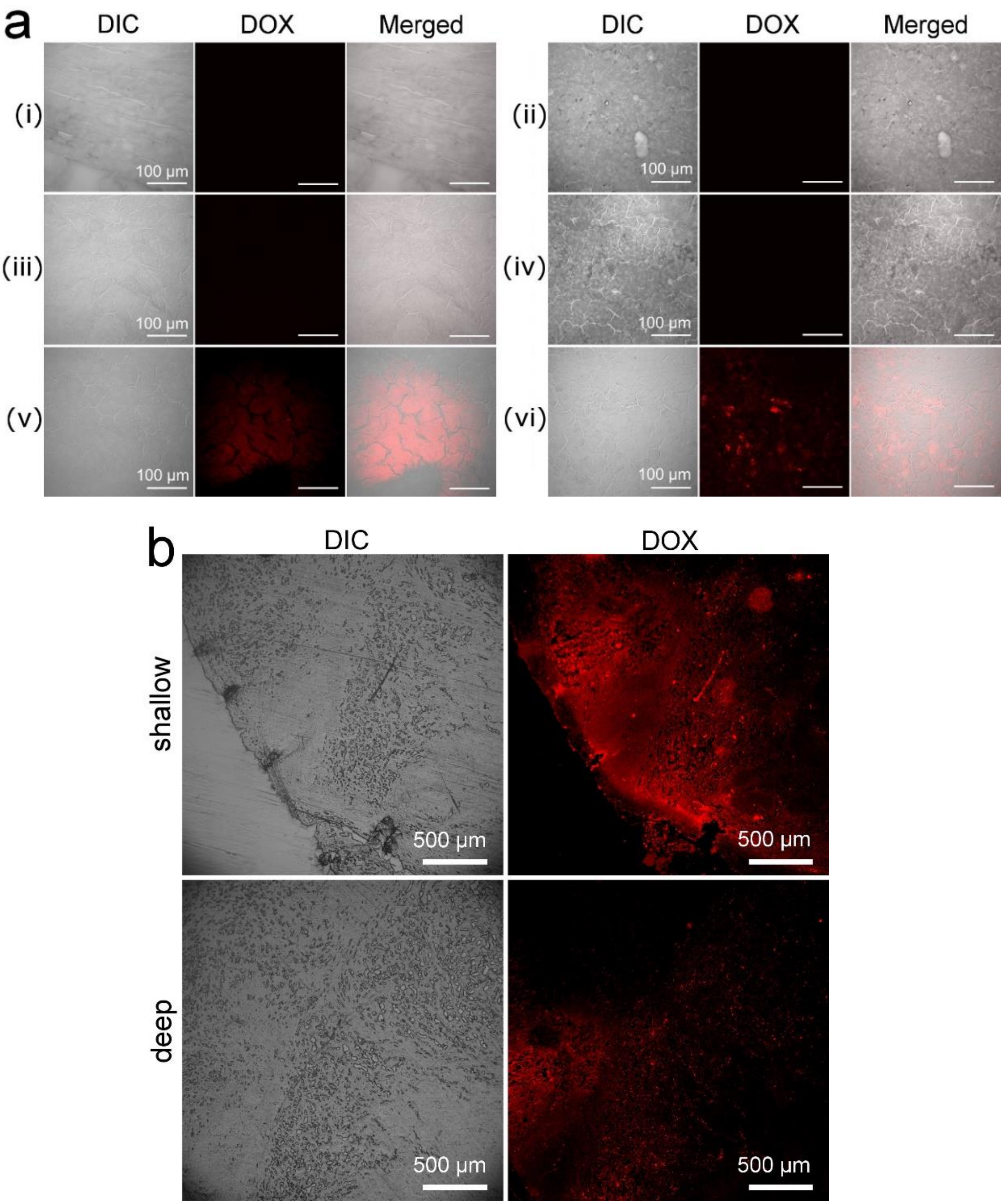

Fig. S11 a Histological fluorescence imaging results of major organs (i, heart; ii, lung; iii, kidney; iv, spleen; v, liver) and (vi) tumor tissue. **b** Fluorescence imaging of shallow (<1.5 mm from the surface) and deep (>2.0 mm deep tumor tissue) tumor tissues, suggesting the efficient penetration of CDSDM NRs especially in shallow tissue. The tumor tissue was about 6 mm in diameter. The imaging was detected based on the DOX fluorescence of NRs.

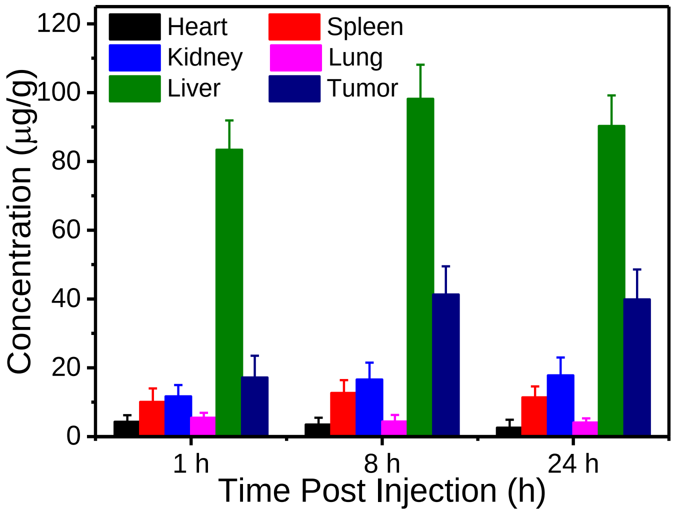

Fig. S12 ICP-MS results of the Si biodistribution in B16F10 tumor-bearing mice after intravenously injected with CDSDM NRs for different times. The percentages of intratumoral Si accumulation were about 13% at 1 h; 23% at 8 h; 24% at 24 h.

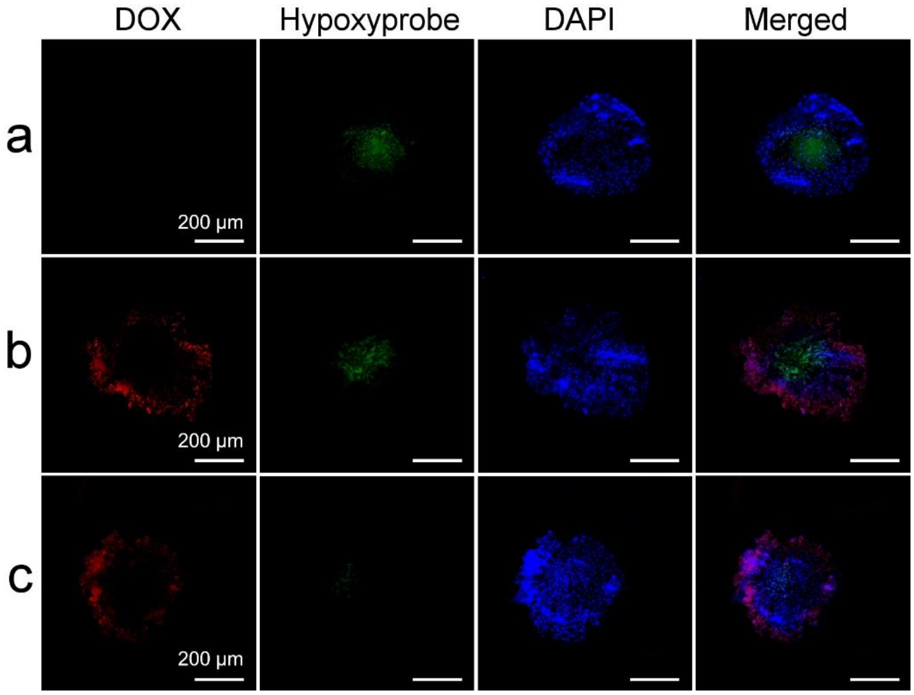

Fig. S13 Hypoxia levels in 3D tumor spheroids after treated with **a** PBS, **b** hDSDM NPs and **c** CDSDM NRs for 6 h. Hypoxia (green fluorescence, detected by commercial Hypoxyprobe) developed in the central regions of tumor spheroids, and as shown, CDSDM NR treatment led to significantly relieved hypoxia. These imaged cryo-sections are the longitudinal median sections of 3D tumor spheroids, and the center of tumor spheroids are relatively blank possibly due to the necrosis of tumor cells.

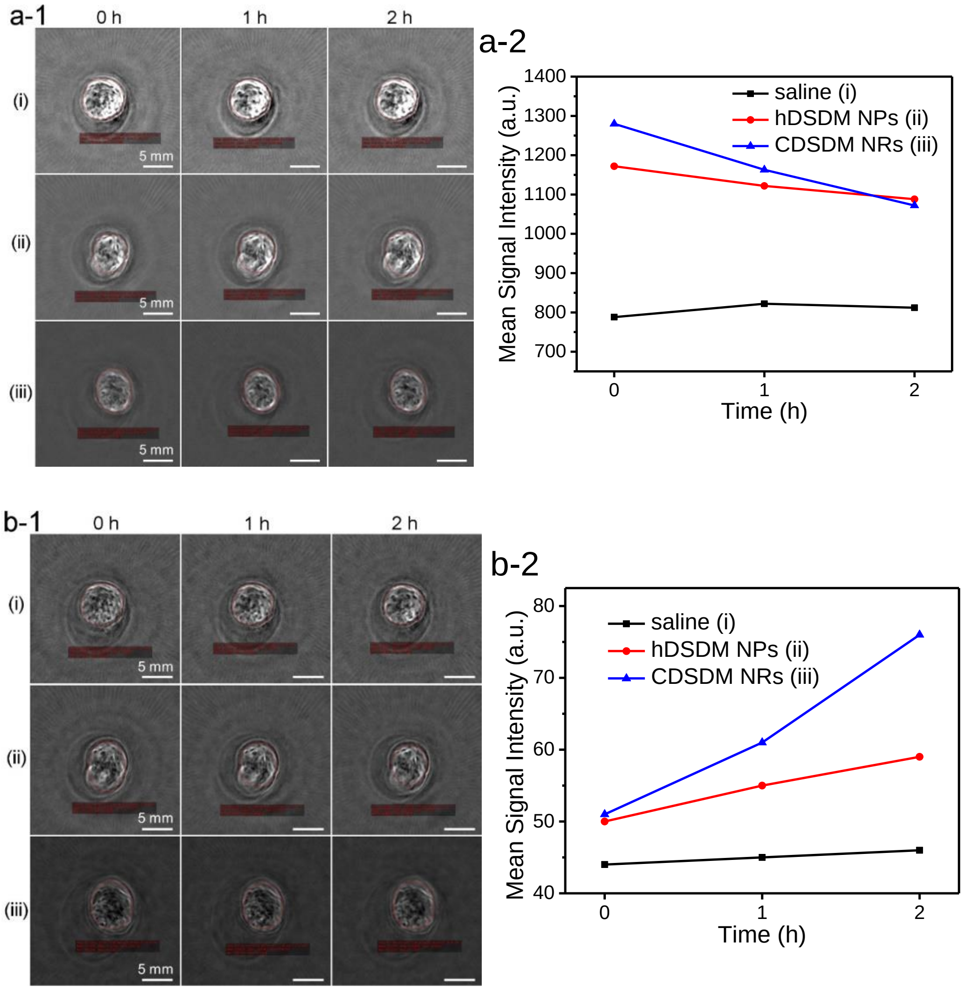

Fig. S14 Photoacoustic imaging of mice tumor tissue that received (i) saline, (ii) hDSDM NP or (iii) CDSDM NR treatment. Measurement was performed using the Endra Nexus128 PA imaging system. The mice were carefully kept anaesthetized so that the same section of each tumor tissue (about 3 mm from tumor surface) could be imaged for better comparison. The signal accumulation depth was set at 1 mm. At each time point, imaging was sequentially performed at **a** 760 nm and **b** 850 nm. The images were exported at default image contrast, and varying image contrast did not change the signal intensity value.

Due to the different peak absorption wavelengths of oxyhemoglobin (~850 nm) and deoxyhemoglobin (~760 nm) [S1], signal enhancement at 850 nm and signal weakening at 760 nm represents the oxygenation in tumor tissue. As shown in Fig. S14, treatment with CDSDM NRs resulted in more obvious signal enhancement (by 32.8%) than hDSDM NPs (by 15.2%) at 850 nm; while at 760 nm, the signal attenuation was more obvious when treated with CDSDM NRs (by 7.2%) than hDSDM NPs (by 16.3%). Therefore, it was concluded that CDSDM NRs could provide more effective oxygenation than hDSDM NPs.

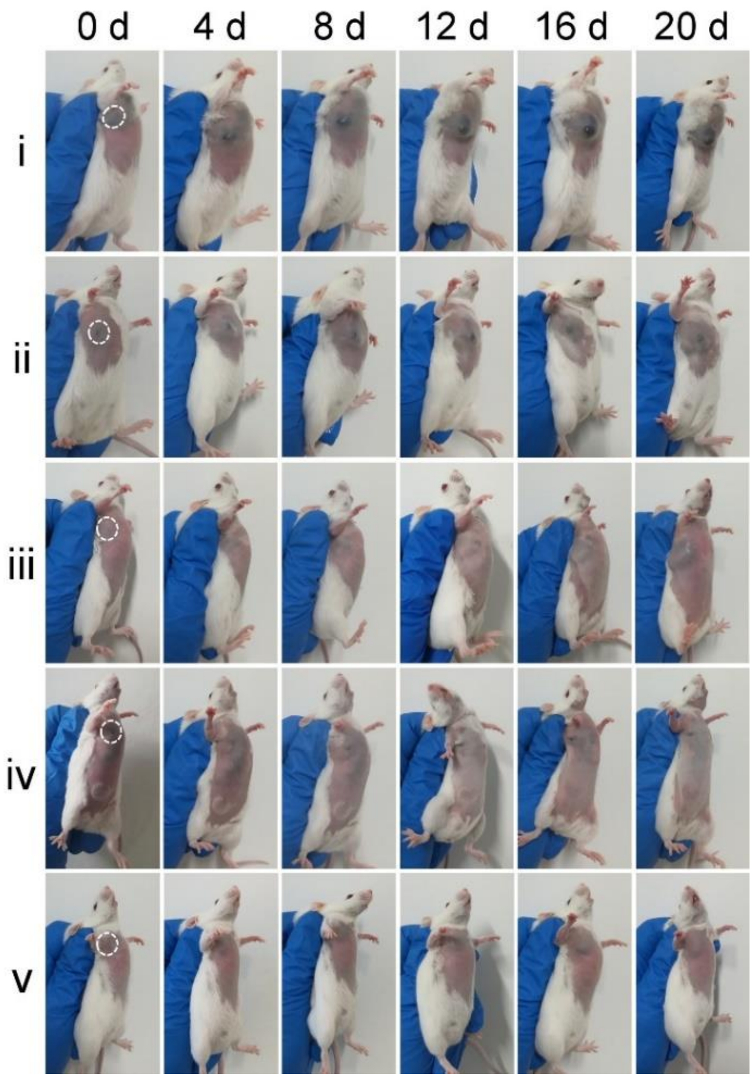

Fig. S15 Photographs of representative mice in each group (i, saline; ii, saline and CTLA-4 blockade; iii, hDSDM NPs and CTLA-4 blockade; iv, CDSDM NRs and CTLA-4 blockade; v, CDSDM/CSDM NRs and CTLA-4 blockade) taken every 4 days during treatment

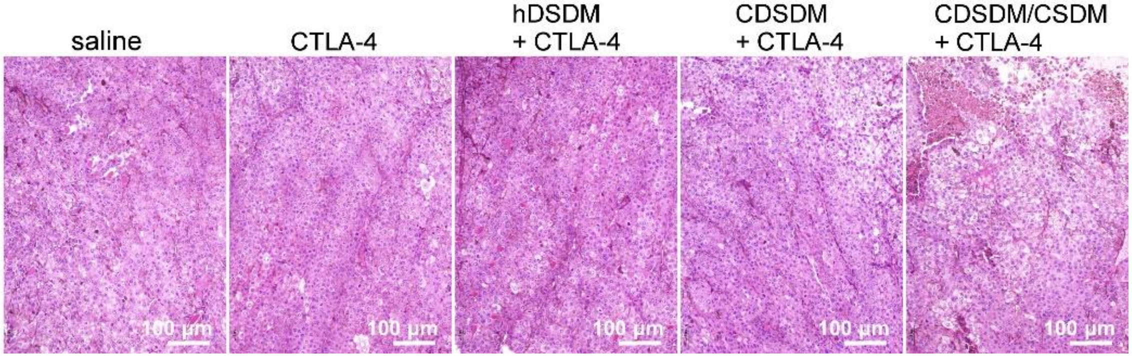

Fig. S16 Representative hematoxylin–eosin stained histopathological sections of tumor tissues from mice of different groups

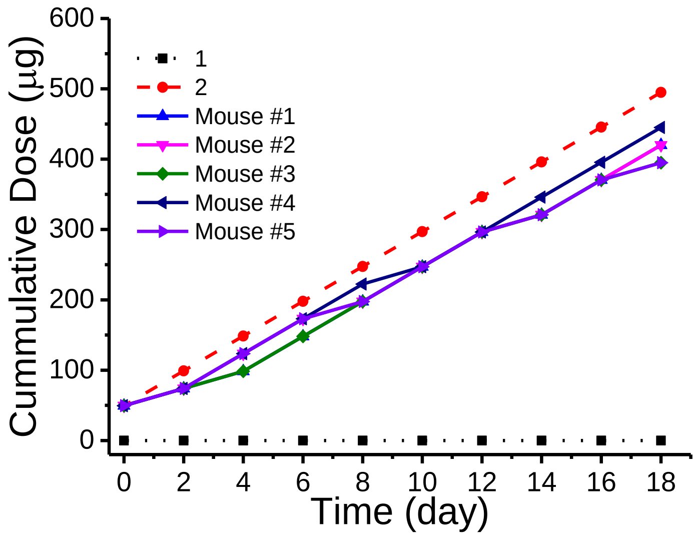

Fig. S17 Cumulative DOX dose received by each mouse from different groups: (1) saline and saline + CTLA-4 blockade groups, (2) hDSDM NPs + CTLA-4 blockade, and CDSDM NRs + CTLA-4 blockade groups, and the CDSDM/CSDM NRs + CTLA-4 blockade group.

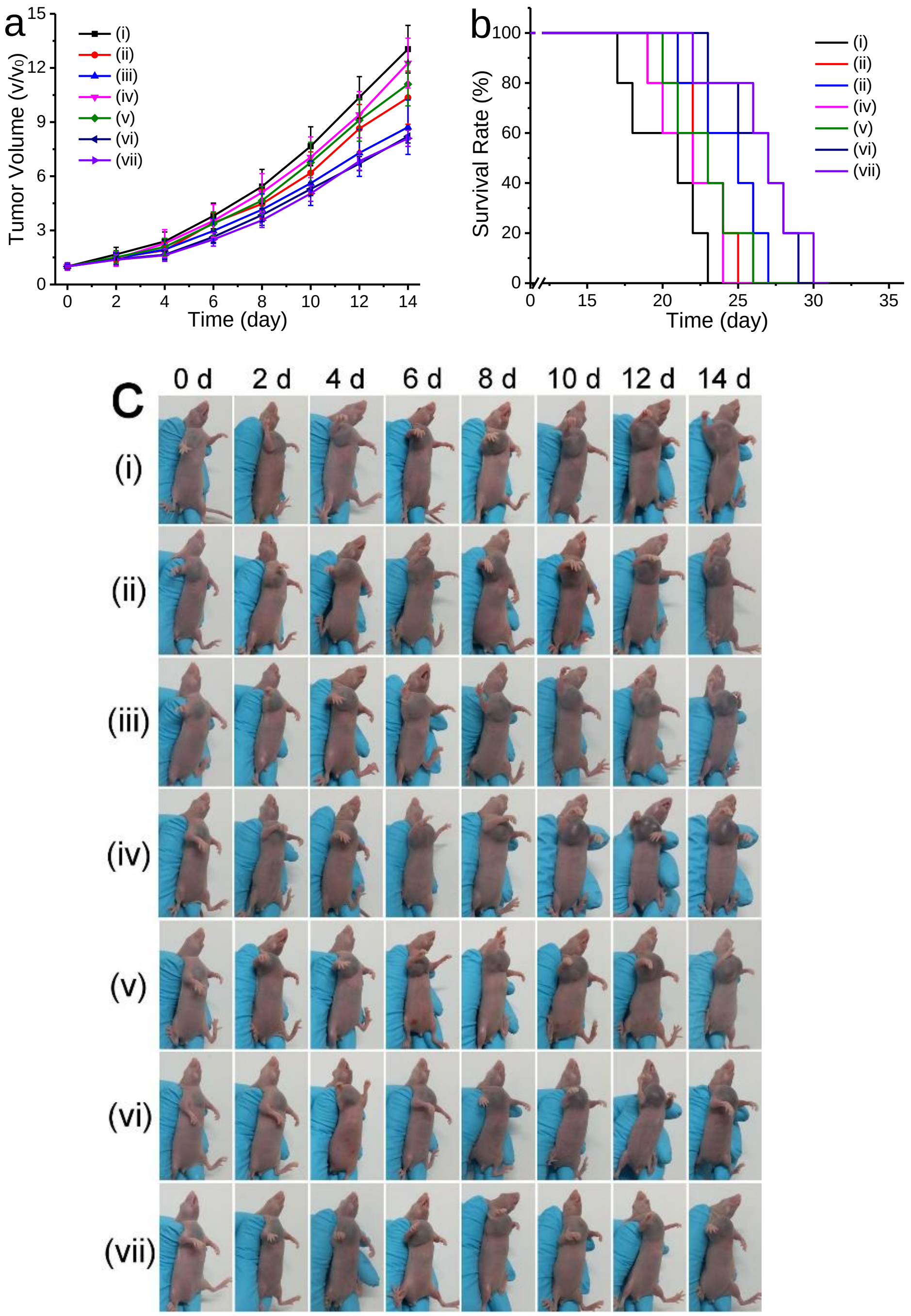

Fig. S18 a B16F10 tumor volume in nude mice that received different treatments (i, saline; ii, CSDM NRs; iii, CDSDM NRs; iv, saline + CTLA-4 blockade; v, hDSDM NPs + CTLA-4 blockade; vi, CDSDM NRs + CTLA-4 blockade; vii, CDSDM/CSDM NRs + CTLA-4 blockade). **b** Survival rate of mice in each group during and after treatment. **c** Photographs of representative mice in each group. No obvious weight loss was observed in each group.

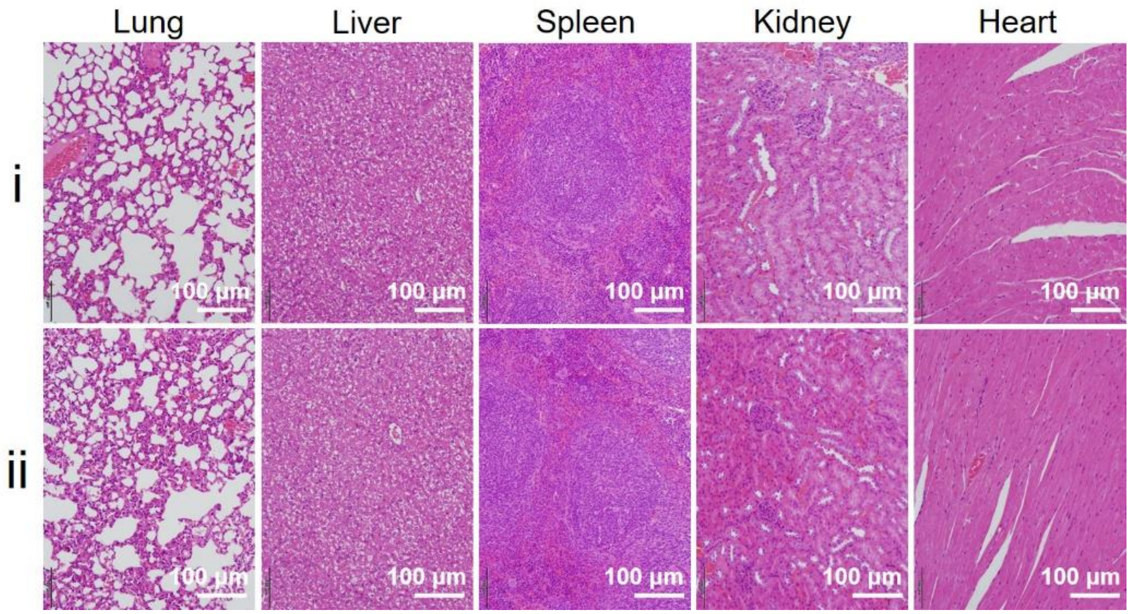

Fig. S19 Representative hematoxylin–eosin stained histopathological sections of major organs from mice (i) treated with saline or (ii) with CDSDM/CSDM NRs (adaptive administration) and CTLA-4 blockade

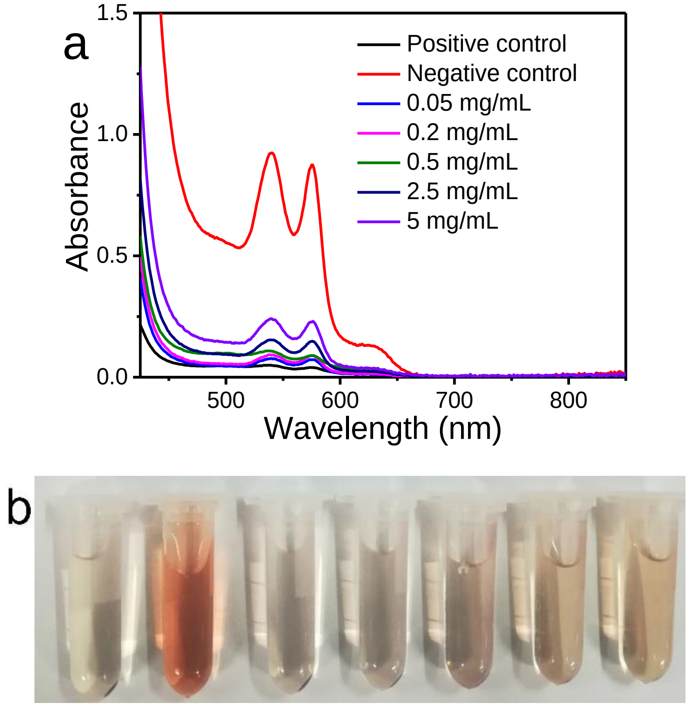

Fig. S20 a UV-Vis absorption spectra and **b** corresponding photograph of hemolysis test of CDSDM NRs on mice. Blood was subjected to centrifugation at 10000 rpm for 5 min to collect red cells, which were then washed with pH 7.4 PBS twice. NPs in PBS were incubated with red cells at different concentrations for 12 h. Incubation with PBS and water were used as positive and negative controls, respectively.

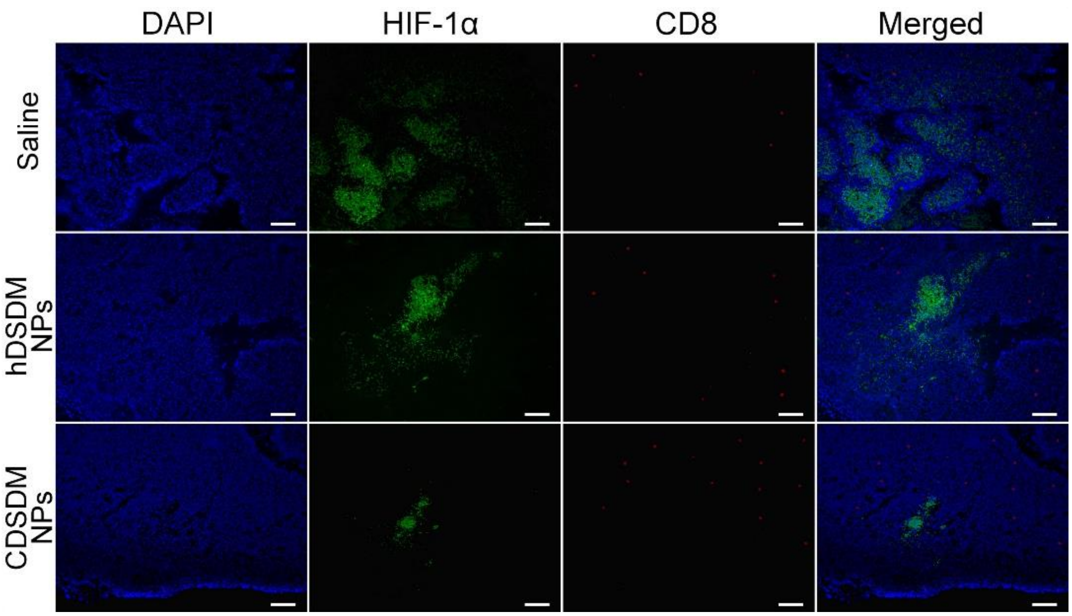

Fig. S21 Immunofluorescence results of the co-staining of HIF-1α and CD8 to shallow (0–1.5 mm from surface) tumor tissues from mice received 4 d of the indicated treatments. Scale bar is 200 μm.

Table S1 Properties of CSDM NRs and CDSDM NRs

	Core diameter	[DOX] in cores[a]	NR diameter	[DOX] in shells	[DOX] in whole NRs
CSDM NRs	90 nm	0	120 nm	10.2 wt%	4.9 wt%
CDSDM NRs	95 nm	9.6 wt%	125 nm	10.2 wt%	9.9 wt%

[a]DOX concentrations were measured via UV-Vis absorption measurement of the released DOX upon core hydrolysis or NR decomposing.

Supplementary References

[S1]F. Cao, Z. Qiu, H. Li, P. Lai, Photoacoustic imaging in oxygen detection. Appl. Sci. **7**(12), 1262 (2017). https://doi.org/10.3390/app7121262
